# Direct Nitridation Synthesis of Quasi-Spherical β-Si_3_N_4_ Powders with CaF_2_ Additive

**DOI:** 10.3390/ma12182870

**Published:** 2019-09-05

**Authors:** Yu Lan, Xiaomin Li, Jinpeng Luo, Lang Zhou, Xiuqin Wei, Chuanqiang Yin

**Affiliations:** Institute of Photovoltaics, Nanchang University, Nanchang 330031, China

**Keywords:** CaF_2_ additive, silicon nitride, direct nitridation, quasi-spherical, powder technology

## Abstract

In this work, the quasi-spherical β-Si_3_N_4_ powders were synthesized via an efficient direct nitridation strategy with CaF_2_ as the catalytic material under NH_3_ atmosphere. The effect of CaF_2_ on phase composition and crystalline morphology was studied. CaF_2_ additive can accelerate the nitridation of silicon powders, and the particles of nitridation products tend to have an equiaxed structure with the CaF_2_ additive increasing. When 4 wt% CaF_2_ additive or more was added, submicron β-Si_3_N_4_ particles with quasi-spherical morphology and eminent crystal integrity were obtained. In contrast, irregular α-Si_3_N_4_ particles appear as the main phase with less than 4 wt% CaF_2_ additive. The growth mechanism of Si_3_N_4_ particles was also discussed. CaxSiyOz liquid phase is crucial in the nitridation of silicon powders with CaF_2_ additive.

## 1. Introduction

β-Si_3_N_4_ has been extensively used in thermally conductive filler of high-performance, thermal interface materials due to its remarkable superiorities, such as high electrical resistivity, low dielectric loss, and high intrinsic thermal conductivity with the theoretical value of 200–300 Wm^−1^K^−1^ [1,2,3]. To improve the device performance, the compaction density of Si_3_N_4_ powders must be as high as possible. Equiaxed structure is beneficial to maximize the compaction density of Si_3_N_4_ powder. Unfortunately, Si_3_N_4_ particles are difficult to transform into spheres, but they tend to grow in specific directions, forming whiskers, fibers, and hexagonal crystals [4,5,6].

Direct nitridation strategy is a simple and efficient method to acquire high purity Si_3_N_4_ powders [7]. This process can be expressed by Equation (1) or (2):

(1) $$3Si\left(s\right)+2N_{2}\left(g\right)\to Si_{3}N_{4}\left(s\right)$$

(2) $$3Si\left(s\right)+4NH_{3}\left(g\right)\to Si_{3}N_{4}\left(s\right)+6H_{2}\left(g\right)$$

In the process, many metals or metal oxides additives, such as Fe, Ni, MgO, CaO, TiO_2_, Cr_2_O_3_, etc. have been reported [8,9,10,11], which act as catalyt for the nitridation of silicon powders. However, these additives cannot facilitate the growth of β-Si_3_N_4_ particles with approximate spherical morphology. Metal fluoride additives, such as CaF_2_, has been reported to synthesize quasi-spherical silicon nitride powders by a carbothermal reduction and nitridation method [12,13]. However few reports discuss the effect of CaF_2_ additive on the nitridation of silicon powders.

In this article, a simple and efficient method of direct nitridation was developed to synthesis quasi-spherical β-Si_3_N_4_ powders with CaF_2_ additive. The β-Si_3_N_4_ particles with quasi-spherical morphology and eminent crystal integrity were obtained at the specific content of CaF_2_ additive. Additionally, the underlying growth mechanism of Si_3_N_4_ particles was also discussed.

## 2. Experimental Procedure

In this work, we chosen Si powders (Jinko Solar Co., Shangrao, China) and CaF_2_ (Bejing Chemical Co., Bejing, China) as raw materials. The CaF_2_ was used as an additive, and the content of CaF_2_ incorporated into the Si powder was set to be 0, 1, 3, 4, 7 wt% relative to the Si powder. To mix the raw materials uniformly, a wet ball milling method was adopted, which was operated at 400 rpm for four hours with agate balls using ethanol as mixing medium. Then, the mixtures were dried at 50 °C in a blast oven. Finally, the processed mixtures were loosely packed into corundum crucible (5 cm × 5 cm × 5 cm) and heated in a tube furnace under ammonia gas (99.999%) at 1300 °C for three hours with the heating rate of 10 °C/min, respectively. 

The produced powders were characterized by X-ray diffraction (XRD, PANalytical, EMPYREAN, Almelo, The Netherlands) using Cu k_α_ radiation (λ = 0.15405 nm). The morphology and microstructure of the powders were examined by field-emission scanning electron microscope (FESEM, JSM-6701F, JEOL, Tokyo, Japan) and high-resolution transmission electron microscopy (HRTEM, JEM-2100F, JEOL, Tokyo, Japan). X-ray photoelectron spectroscopy (XPS) analysis was carried out using ESCALAB 250Xi (Thermo Fisher, MA, USA) instrument. 

## 3. Results and Discussion

**Figure 1:**
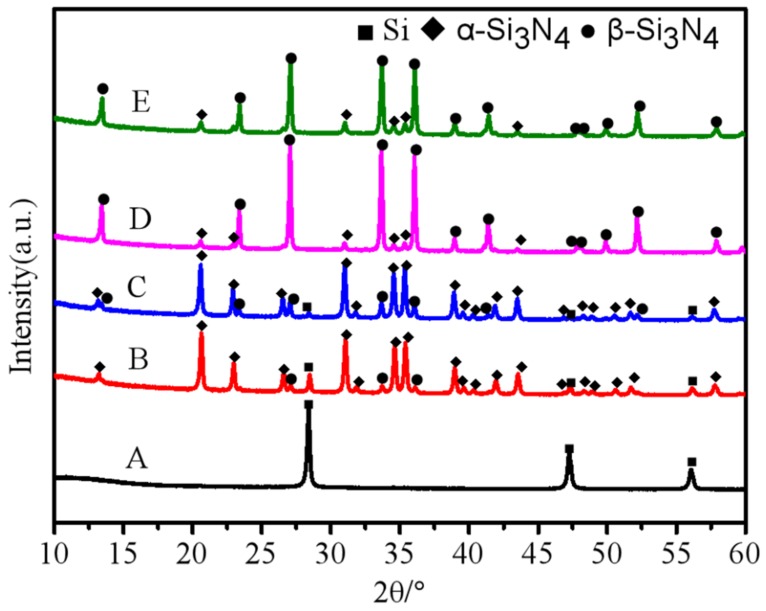
X-ray diffraction patterns of the products obtained at 1300 °C under NH_3_ atmosphere with different contents of CaF_2_ additive (A) 0 wt% CaF_2_; (B) 1 wt% CaF_2_; (C) 3 wt% CaF_2_; (D) 4 wt% CaF_2_; (E) 7 wt% CaF_2._

The XRD patterns of the obtained nitridation products are shown in Figure 1. Only the silicon phase with a cubic structure exists in the sample without CaF_2_ additive, and no Si_3_N_4_ characteristic peaks are detected in the detection limit of X-ray diffraction. Under different contents of CaF_2_ additive, nitridation reaction was strongly accelerated. When 1 wt% CaF_2_ was added, Si characteristic peaks became weaker and Si_3_N_4_ phase was the main composition. With an increased amount of the CaF_2_ additive, the residual silicon content in the products decreased. When 4 wt% CaF_2_ or more was added, silicon powders were completely nitride, and the Si characteristic peaks were not detected by XRD analysis. Additionally, no trace impurities were detected in the as-obtained nitridation products, such as Si_2_N_2_O, Ca compounds and fluoride. One possible explanation why CaF_2_ characteristic peaks were not detected in the XRD patterns is that when the crystal interplanar spacing of CaF_2_ (111) is similar to Si (111), CaF_2_ (111) characteristic peak may be hidden in Si (111) diffraction peak. Secondly, the CaF_2_ additive may have evaporated during or after the reaction process [14]. Thirdly, the CaF_2_ catalyst is likely to react with oxidation layer on the Si particles to form the trace phase. All of these may explain why CaF_2_ peaks could not be detected in the XRD patterns above. As seen in Figure 1, the obvious crystal form transformation occurred in the specific content of CaF_2_ additive. A slow-heating rate is vital in obtaining high purity α-Si_3_N_4_, especially in the nitridation process of pure silicon powders without any diluent. However, with CaF_2_ additive, the nitridation reaction could occur at a fast-heating rate of 10 °C/min under NH_3_ atmosphere. This may be a result of the catalytic effect of CaF_2_ that reacts with SiO_2_ on the Si particle surface as follows [15,16].

(3) $$2CaF_{2}\left(s\right)+SiO_{2}\left(s\right)\to SiF_{4}\left(g\right)+2CaO\left(s\right)$$

The fresh silicon surface was exposed in the NH_3_ atmosphere and the nitridation reaction was accelerated due to the removal of oxide layer on the Si particle surface by CaF_2_ additive. Meanwhile, the content of β-Si_3_N_4_ phase increased with the increased amounts of the CaF_2_ additive owing to the reaction of CaO from reaction (3) with SiO_2_ on the Si particles surface, which urges the formation of Ca_x_Si_y_O_z_ liquid. The process can be established as the following equation.

(4) $$ySiO_{2}\left(s\right)+xCaO\left(s\right)\to Ca_{x}Si_{y}O_{z}\left(l\right)$$

It is preferential to form β-Si_3_N_4_ phase when enough CaxSiyOz liquid exists in the reaction system [7]. The content of Ca_x_Si_y_O_z_ liquid and β-Si_3_N_4_ increase with increased CaF_2_ additive. This tendency can be seen from the X-ray diffraction patterns.

**Figure 2:**
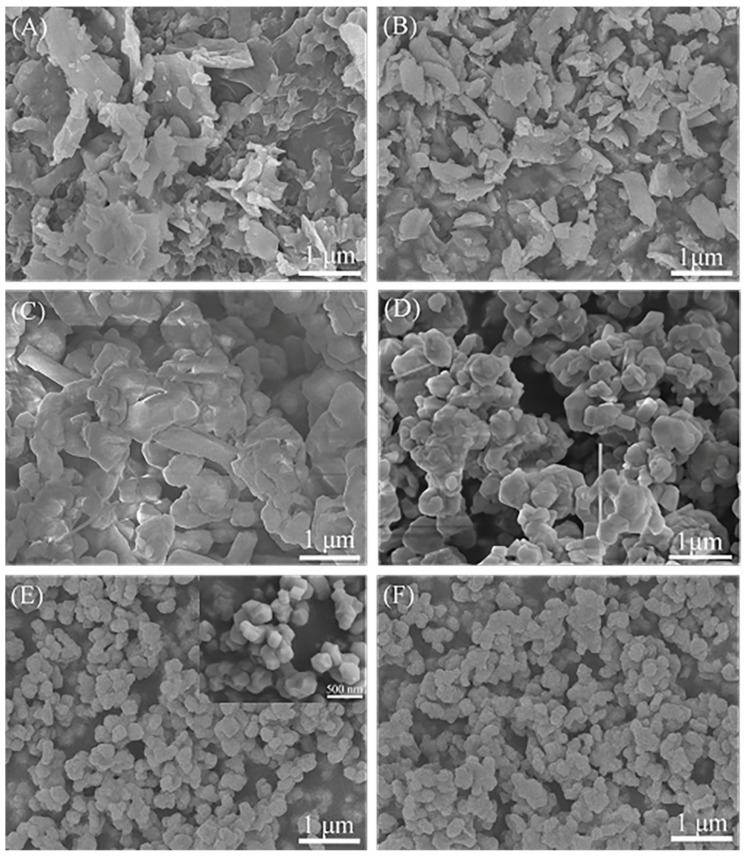
Scanning electron microscope images of the raw silicon (**A**) and the nitridation products obtained at 1300 °C under NH_3_ atmosphere with different contents of CaF_2_ additive (**B**) 0 wt% CaF_2_; (**C**) 1 wt% CaF_2_; (**D**) 3 wt% CaF_2_; (**E**) 4 wt% CaF_2_; (**F**) 7 wt% CaF_2._

Figure 2 shows SEM images of nitrification products with different CaF_2_ additive contents as well as the the morphology of raw silicon powders, provided as a reference. The morphology of the raw Si particles is irregular flake, as shown in Figure 2A. The morphology of the sample without CaF_2_ additive is also irregular flake, which is similar to the morphology of the raw Si particles. In combination with the XRD analysis, the Si particles have obviously not been nitrided under this experiment condition. However, with the addition of CaF_2_ additive, the shape of the as-obtained Si_3_N_4_ particles gradually evolve into relatively smooth edges, smaller particle size, and approximately equiaxed structure, as shown in Figure 2. The morphologies of the samples with 1 wt% and 3 wt% of CaF_2_ additive present various shapes, including columnar crystals, whiskers, and irregular particles with sharp edges. Additionally, serious aggregates are discovered in the powders, as shown in Figure 2C,D. The decisive effect of SiO gas can be seen on the formation of the silicon nitride whisker, which originated from the SiO_2_ on the surface of Si particles at a high temperature with NH_3_ gas. This process can be described as the following equations [7]:

(5) $$SiO_{2}\left(s\right)+Si\left(s\right)\to 2SiO\left(g\right)$$

(6) $$SiO\left(g\right)+NH_{3}\left(g\right)\to Si_{3}N_{4}\left(s\right)+H_{2}O\left(g\right)$$

In addition, sharp edges indicate that Si3N4 crystals tend to grow in specific directions. Surprisingly, In the sample with 4 wt% CaF2 or more, it is mainly spherical particles that can be seen in Figure 2E,F and the insert of Figure 2E. Slight aggregates of the submicron particles were also discovered. This phenomenon can be explained by the theory of crystal growth: Crystal tends to grow into a structure with low surface energy [17]. The formation of quasi-spherical structure is due to the existence of Ca_x_Si_y_O_z_ liquid phase in the reaction system. In the case of insufficient Ca_x_Si_y_O_z_ liquid phase, the columnar structure of Si_3_N_4_ particles is expected to be aligned along the low-index crystallographic direction [12]. However, in the case of the abundant Ca_x_Si_y_O_z_ liquid, the Si_3_N_4_ particles tend to grow along the lowest energy direction of the solid-liquid interface. Accordingly, the Si_3_N_4_ particles grow into a quasi-spherical morphology.

**Figure 3:**
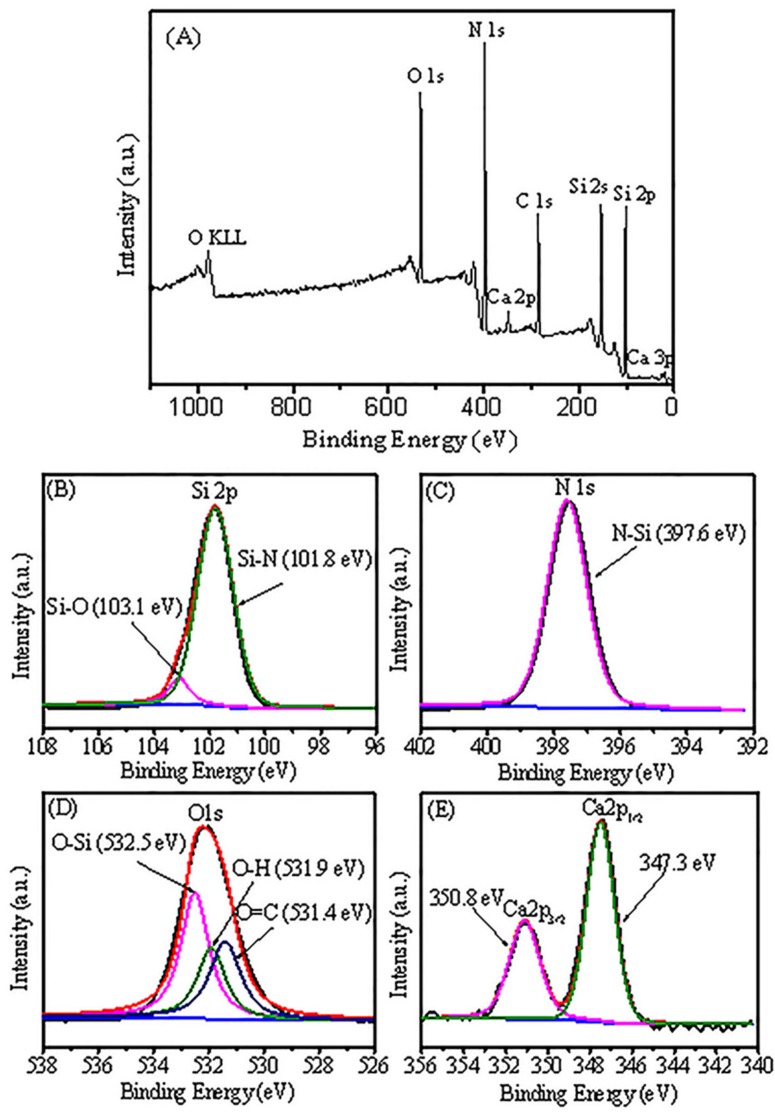
X-ray photoelectron spectroscopy survey (**A**) and X-ray photoelectron spectroscopy spectra of Si (**B**), N (**C**), O (**D**), and Ca (**E**) elements in the Si_3_N_4_ particles obtained at 1300 °C under NH_3_ atmosphere with 4 wt% of CaF_2_ additive.

The surface composition and bonding structure of the obtained product of the 4 wt% CaF_2_ additive were determined by XPS test, as shown in Figure 3. It can be seen from the full spectrum of XPS that Si, O, N, and Ca are the main components in the product. The Si and N elements originate from Si_3_N_4_, the Ca and O elements come from the Ca_x_Si_y_O_z_ liquid phase. The F element is not detected by XPS, because F volatilizes in the form of SiF_4_ gas. Figure 3B–E depict the high-resolution XPS scans of Si2p, N1s, O1s, and Ca^2+^. Through peak separation, the Si2p can be divided into two peaks at 101.8 eV and 103.1 eV; the peak at 101.8 eV represents Si-N bond, while the peak at 103.1 eV is attributed to Si-O bond. The Si-O bond may be derived from Ca_x_Si_y_O_z_ liquid phase. The 397.6 eV peak of N1s corresponds to the N-Si bond. The O1s is divided into three peaks, 531.4 eV, 531.9 eV, and 532.5 eV, respectively. The first two peaks are attributed to CO_2_ and H_2_O in the test environment, respectively, and the 532.5 eV peak is attributed to the O-Si bond. The two peaks in the Ca2p spectrum correspond to Ca2p3/2 with a bond energy of 347.3 eV and Ca2p1/2 with a bond energy of 350.8 eV. The two peaks are assigned to oxygen-bonded calcium, which means Ca is mainly present in the surface oxide layer. These results indicate that an oxidation phase containing Si and Ca elements exists in the as-obtained nitridation products.

**Figure 4:**
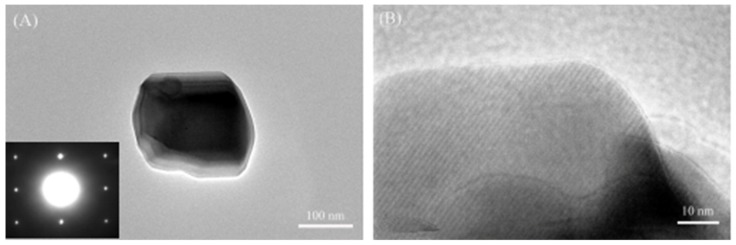
(**A**) Transmission electron microscopy, (**B**) high-resolution transmission electron microscopy image of a typical Si_3_N_4_ particle obtained at 1300 °C under NH_3_ atmosphere with 4 wt% of CaF_2_ additive.

Further details about the morphology and crystal structure of the typical Si_3_N_4_ particle in the sample synthesized with 4 wt% CaF_2_ additive can be revealed by TEM and HRTEM, as shown in Figure 4. The Si_3_N_4_ particle exhibits submicron quasi-spherical morphology and eminent crystal integrity. As seen in the inset of Figure 4A, the selected-area electron diffraction (SAED) pattern and HRTEM image indicate the particles synthesized with 4 wt% CaF_2_ additive are typical single-crystal β-Si_3_N_4_, and there was no obvious amorphous oxide layer on the particle surface. However, the oxidation phase containing Si and Ca elements exists in the nitridation product measured by XPS. The possible reason is the existence of Ca_x_Si_y_O_z_ phase between silicon nitride particles facilitated the particles aggregation through liquid bond, as shown in Figure 2E,F.

## 4. Conclusions

In this paper, submicron β-Si_3_N_4_ powders with quasi-spherical morphology were prepared through direct nitridation synthesis under NH_3_ atmosphere at 1300℃ with appropriate content of CaF_2_ additives (4 wt% or more). CaF_2_ additive can accelerate nitridation of silicon powders and its content has a great impact on the formation of Si_3_N_4_ particles. The SEM images reveal that the morphology of Si_3_N_4_ particle transforms to spherical with the CaF_2_ additive increasing. It contributes to form quasi-spherical β-Si_3_N_4_ particles due to the formation of Ca_x_Si_y_O_z_ liquid phase. The SAED and HRTEM images indicate that the β-Si_3_N_4_ particles are single crystal and almost without an amorphous oxidation layer. The phase and morphology of Si_3_N_4_ particles can be controlled through the adjustment of the content of CaF_2_ additive. This preparation offers a very convenient and meaningful way to prepare quasi-spherical β-Si_3_N_4_ powders and is also suitable for industrial application.
